# JMT103 versus Non‐Denosumab or Denosumab Treatment in Chinese Patients with Unresectable or Surgically Challenging Giant Cell Tumor of Bone: A Propensity Score‐Matched Comparison

**DOI:** 10.1002/cam4.71340

**Published:** 2025-11-25

**Authors:** Hairong Xu, Yong Zhou, Feng Wei, Yi Ding, Huachao Shan, Yongkun Yang, Weifeng Liu, Tao Jin, Yi Luo, Fan Tang, Minxun Lu, Xin He, Wenli Zhang, Shaomin Yang, Lihua Zhang, Juan Wang, Hong Li, Chongqi Tu, Xiaohui Niu

**Affiliations:** ^1^ Department of Orthopedic Oncology Surgery Beijing Jishuitan Hospital, Capital Medical University Beijing China; ^2^ Department of Orthopedics West China Hospital, Sichuan University Chengdu China; ^3^ Department of Medical Oncology and Radiation Sickness Peking University Third Hospital Beijing China; ^4^ Department of Pathology Beijing Jishuitan Hospital, Capital Medical University Beijing China; ^5^ Department of Pathology West China Hospital, Sichuan University Chengdu China; ^6^ Department of Pathology Peking University Third Hospital Beijing China; ^7^ Department of Radiology Peking University Third Hospital Beijing China; ^8^ CSPC Pharmaceutical Group Limited Shijiazhuang China

**Keywords:** effectiveness, giant cell tumor of bone, JMT103, propensity score analysis, safety

## Abstract

**Background:**

Giant cell tumors of bone (GCTB) are RANK/RANK‐ligand positive, progressive osteolytic tumors. There was no medical treatment for GCTB based on efficacy and safety data from Chinese patients. A single‐arm, phase II study demonstrated the promising efficacy of JMT103 in unresectable or surgically challenging GCTB. Patient‐level outcomes from the single‐arm trial were compared with real‐world data from denosumab or non‐denosumab treated patients to estimate comparative efficacy in unresectable or surgically challenging GCTB.

**Methods:**

The real‐world data were retrospectively collected from three hospitals between January 2013 and December 2023. The eligibility criteria of the two cohorts were based on the JMT103 single‐arm study. Propensity score matching was used to balance the baseline characteristics of patients in the JMT103 and the real‐world cohorts. The primary endpoint was histopathological or 12 week radiological objective tumor response rate (OTR). Secondary endpoints included tumor response rate throughout the study, objective response rate, disease control rate, and safety profiles.

**Results:**

166 and 135 patients were finally included in the non‐denosumab and denosumab cohorts, respectively. After 1:1 nearest neighbor matching, the OTR of the JMT103 cohort was significantly higher than that of the non‐denosumab cohort (94.2% vs. 4.8%), and was comparable with that of the denosumab cohort (92.0% vs. 67.0%). The same results were observed in tumor response rate throughout the study (JMT103 vs. non‐denosumab: 94.2% vs. 4.8%, JMT103 vs. denosumab: 94.3% vs. 86.4%), objective response rate (83.5% vs. 11.1%, 87.4% vs. 80.0%), and disease control rate (100% vs. 70.4%, 100.0% vs. 98.8%).

**Conclusion:**

JMT103 has manageable safety profiles with better effectiveness than non‐denosumab and a trend toward greater effectiveness than denosumab in patients with unresectable or surgically challenging GCTB.

**Trial Registration:**

NCT05402865, NCT04255576

## 1 Introduction

Giant cell tumor of bone (GCTB) is a rare, osteolytic tumor with an annual incidence rate of one to three per million persons in the Chinese population, characterized by local aggressiveness and a high risk of local recurrence [1, 2]. It commonly occurs in patients aged 20–40 years [3], accounting for 5%–10% of all primary bone tumors. In 2020, the World Health Organization defined GCTB as an intermediate malignant tumor [4], which causes bone destruction, severe pain, pathologic fractures, and occasional metastatic spread [5].

Denosumab, an anti‐RANKL antibody (IgG2 subtype), inhibits osteoclast differentiation and function, and bone matrix absorption by blocking the RANKL/RANK signaling pathway [6, 7]. It showed long‐term disease control and provided other benefits for patients with GCTB, including reduced pain requiring less analgesia and improved function, mobility, and bone repair [8, 9, 10, 11]. Therefore, it has been recommended to treat unresectable or recurrent metastatic GCTB or to reduce the difficulty of surgery before operation [12]. While the long‐term preoperative application of denosumab in patients with GCTB has been found to increase the enhancement rate of the lesion, thus making it difficult to achieve a clear margin during surgery and, therefore, increasing the risk of recurrence [13, 14].

JMT103 is a novel, fully humanized IgG4 monoclonal antibody targeting RANKL. By replacing the Fc fragment of denosumab from immunoglobulin IgG2 to IgG4, JMT103 targeted RANKL with higher affinity and specificity than that of denosumab [15]. A single‐arm, phase II study was conducted to explore the efficacy and safety of JMT103 in patients with unresectable GCTB and demonstrated that JMT103 had a rapid onset of action (median 0.95 months), a high objective tumor response rate (93.5%), and great improvement in quality of life in patients with unresectable GCTB [16]. Besides the encouraging anti‐tumor efficacy, JMT103 also showed a manageable safety profile in the previous trials [17, 18].

Although denosumab was approved for the treatment of unresectable GCTB in China in May 2019, it has been granted conditional market approval due to the lack of efficacy and safety profiles in Chinese patients with GCTB, so when this phase II JMT103 study was conceptualized and initiated in early 2020, there was still no medical treatment for unresectable GCTB based on efficacy and safety data from Chinese patients and unmet medical needs were urgently addressed. A head‐to‐head comparison of JMT103 with denosumab in Chinese patients with GCTB has not been conducted yet. China has a high prevalence of GCTB, where it accounts for 20% of primary benign skeletal tumors [2], while the data from Chinese patients about the treatment of GCTB are limited. Therefore, we designed and conducted this study to better understand the effectiveness of denosumab and non‐denosumab treatment (other anti‐GCTB drugs other than denosumab or no GCTB medications) in real‐world Chinese patients with GCTB and provide NMPA with evidence for decision‐making.

In this study, we compared the outcome of JMT103 from the above single‐arm, phase II study with those receiving non‐denosumab or denosumab treatment in real‐world settings.

## 2 Methods

### 2.1 JMT103 Cohort From Single‐Arm, Phase II Study

Full details of the JMT103 single‐arm, phase II study (NCT04255576) were described elsewhere [16]. The key eligibilities included patients (age ≥ 18 years) with historically confirmed unresectable GCTB or surgical‐challenging GCTB (defined as surgical resection likely to result in severe morbidity), and ECOG of 0–2. This trial was conducted in 30 hospitals in China. One hundred thirty‐nine patients were enrolled from May 2020 to June 2021. Patients received subcutaneous injections of JMT103 at 2 mg/kg (loading doses on day 8 and day 15 for the first 4 weeks) every 4 weeks until disease progression, complete resection, intolerable toxicity, withdrawn consent, or absence of benefit from JMT103 treatment (whichever occurred first). Tumor response was assessed based on inverse Choi density/size (ICDS) criteria every 4 weeks for the first 12 weeks, every 12 weeks until 48 weeks, and every 24 weeks after that until disease progression or based on European Organization for Research and Treatment of Cancer (EORTC) criteria every 12 weeks until disease progression. The study protocol was approved by Independent Ethics Committees at all study sites (site list shown in Table S1). Written informed consent was obtained from all patients before enrollment.

### 2.2 Real‐World Non‐Denosumab or Denosumab Treatment Cohorts

The real‐world data were retrospectively collected from electronic health records between 1 January 2013 and 31 December 2021 of three study sites (Beijing Jishuitan Hospital, Peking University Third Hospital, and West China Hospital of Sichuan University) (NCT05402865). The denosumab cohort comprised GCTB patients treated with denosumab. The non‐denosumab cohort consisted of patients who received medications other than denosumab and no GCTB medications. Patients in the denosumab and non‐denosumab cohorts met the key eligibility criteria of the JMT103 single‐arm phase II study and had at least one imaging or histological evaluation before and after treatment. Key exclusion criteria were known metabolic bone disease before treatment (such as hypoparathyroidism, hyperparathyroidism, hyperthyroidism, hypothyroidism, hypopituitarism, hyperprolactinemia, Cushing syndrome, acromegaly, and Paget disease); co‐occurrence of malignancies including the malignant transformation of GCTB and under anti‐tumor therapy, and patients with no verifiable records. The study protocol and data collection sheet were approved by Independent Ethics Committees at each study site (Beijing Jishuitan Hospital, Peking University Third Hospital, and West China Hospital of Sichuan University).

### 2.3 Outcome Measures

The primary endpoint was the histopathological or 12 week radiological objective tumor response rate (OTR) based on best response, defined as the proportion of the patients with an elimination of at least 90% of osteoclast‐like giant cells relative to baseline or radiologic determination of tumor response (complete response + partial response) within 12 weeks assessed with ICDS or EORTC criteria. The key secondary endpoint was tumor response rate throughout the study, defined as the proportion of the patients with an elimination of at least 90% of osteoclast‐like giant cells relative to baseline or radiologic determination of tumor response (complete response + partial response) throughout the study assessed with ICDS or EORTC criteria. Other secondary endpoints included objective response rate, defined as the percentage of patients who had achieved complete response and partial response, disease control rate, defined as the percentage of patients who had achieved complete response, partial response and stable disease, and safety profiles.

### 2.4 Data Collection

Data collected included demographics, disease characteristics, details of anti‐GCTB treatment during the prespecified period, or histological evaluation before and after the treatment (tumor assessment interval ≥ 14 days), adverse events, and concomitant medication.

### 2.5 Statistical Analyses

All the statistical analyses were performed with SAS software (9.4 version). The sample size was determined based on the model robustness and hypothesis power [19, 20, 21], and 306 of sample size was required from the real‐world study (Supporting Information).

### 2.6 Propensity Score Matching and Adjustment Strategy

Propensity score modeling was constructed by using a binary nonconditional logistic regression method to predict the use of treatment [22]. The selection of covariates was mainly based on the current knowledge of risk or prognostic factors in the management of GCTB [23] and confounders were identified based on backdoor criterion using directed acyclic graphs [24].

Propensity score was used to adjust for differences between patients in the JMT103 and non‐denosumab or denosumab cohorts via 1:1 nearest neighbor matching with a caliper width of 0.2 standard deviations of the logit of the propensity scores [25]. A standardized difference of more than 10% might indicate a significant imbalance in the covariates between cohorts [25].

### 2.7 Propensity Score Matching

The OTR in each cohort was evaluated and the 95% confidence interval (CI) for OTR was calculated with the Wilson score method.

After 1:1 propensity score matching, Pearson's chi‐squared test or Fisher's exact test was used to compare the JMT103 and non‐denosumab cohorts; no statistical test was used to compare the JMT103 and the denosumab cohort, The 95% CI for the rate difference between JMT103 and non‐denosumab or denosumab cohorts was calculated with the Newcombe method. Nonconditional logistic regression was used to calculate the odds ratio (OR) and 95% CI.

### 2.8 Sensitivity Analysis and Subgroup Analysis

Sensitivity analyses were performed to determine the robustness of the primary endpoint outcome by using inverse probability weighting to balance the cohorts for the primary endpoint (analysis 1) by excluding the patients with no postbaseline histopathological evaluation and radiologic examination within 12 weeks (analysis 2), by using a logistics regression model with adjustment for the confounders and potential heterogeneous factors (analysis 3), and by quantitative bias analysis for unknown and unmeasured confounders reported by E‐value (analysis 4).

Exploratory subgroup analyses were performed for the primary endpoint by gender (male vs. female), age (< 40 years vs. ≥ 40 years), metastasis (yes vs. no), relapse (yes vs. no), ECOG performance score (0 vs. ≥ 1) and disease status (surgically challenging vs. unresectable). The Wilson score method was used to calculate 95% confidence intervals for proportions of each subgroup of patients who achieved tumor response. A subgroup analysis would not be conducted if there was a small number of patients (less than 10) per subgroup.

## 3 Results

### 3.1 Patients Characteristics

**FIGURE 1:**
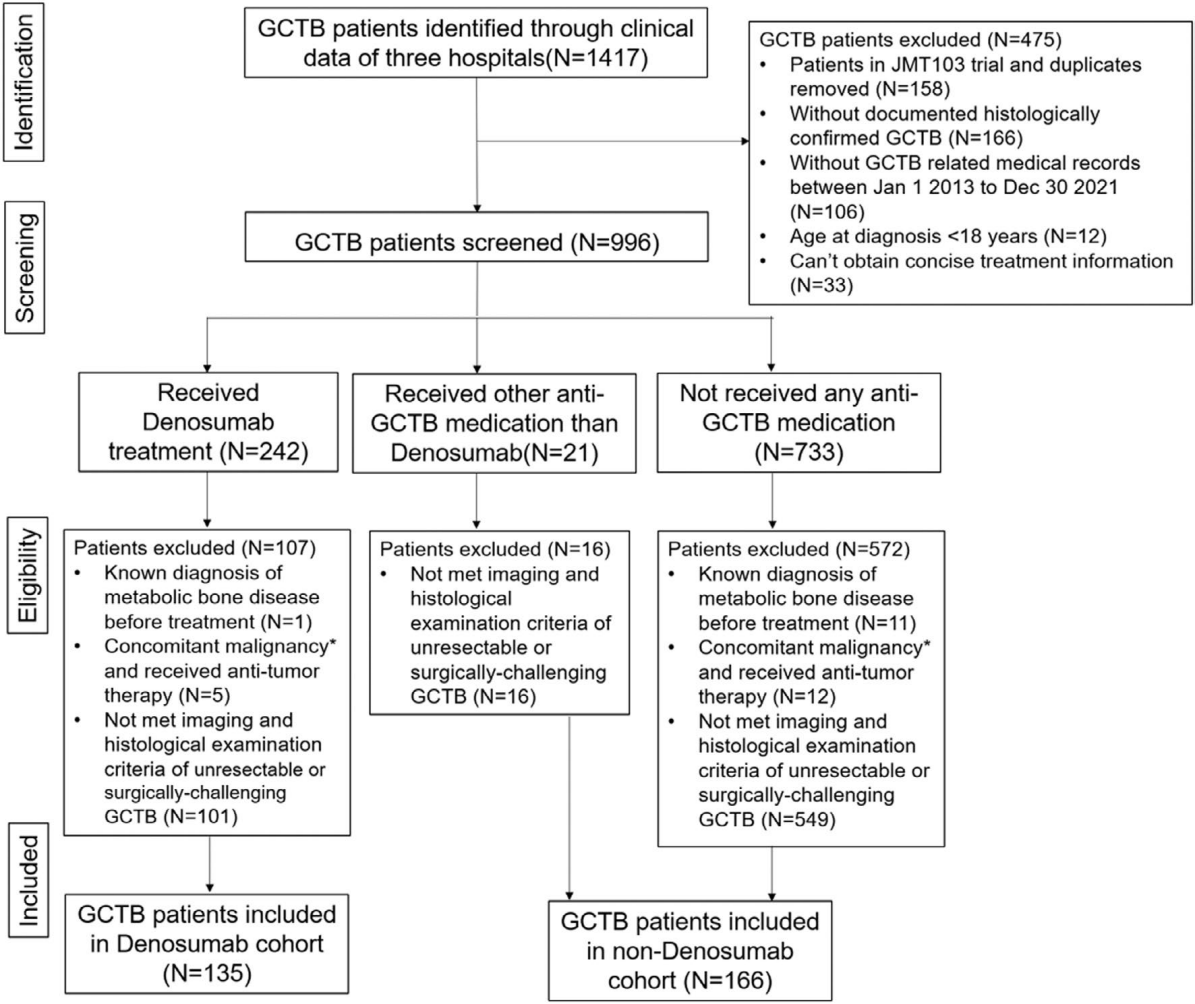
Flow diagram chart showing the identification, screening, eligibility and included process of non‐denosumab and denosumab cohorts. A total of 996 patients with GCTB were screened, and ultimately 135 patients treated with denosumab (denosumab cohort) and 166 patients not treated with denosumab (non‐denosumab cohort) were included. *Including malignant transformation of GCTB. GCTB, Giant cell tumor of bone.

A total of 1417 patients with GCTB were identified in the electrical clinical database of three hospitals, after excluding step‐by‐step due to not meeting the eligibility criteria of the real‐world study, 166 and 135 patients were finally included in the non‐denosumab and denosumab cohorts (Figure 1). Of 166 patients in the non‐denosumab cohort, 27 patients had radiological results within 12 weeks, 142 patients had histopathological results, and 161 patients had either result. Of 135 patients in the denosumab cohort, 98 patients had radiological results within 12 weeks, 37 patients had histopathological results, 114 patients had either result. Of 138 patients enrolled in the JMT103 cohort, 137 patients had radiological results within 12 weeks, 66 patients had histopathological results, and 138 patients had either result.

The demographic and clinical characteristics of non‐denosumab and denosumab treatment patients vary compared with the JMT103 cohort (Table 1 and Table S2). There was a shorter duration of GCTB, a few patients having metastasis or relapse in the non‐denosumab cohort, and more patients having relapse in the denosumab cohort. Of 166 patients in the non‐denosumab cohort, 100% of patients had previous surgery, 12.7% received bisphosphonates, and 87.3% received no GCTB‐specific medication.

**TABLE 1:** Demographics and clinical characteristics of patients in non‐denosumab or denosumab cohort and JMT103 cohort after propensity score matching.

Characteristic	Non‐denosumab cohort (*N* = 104)	JMT103 cohort (*N* = 104)	Standard difference	Denosumab cohort (*N* = 88)	JMT103 cohort (*N* = 88)	Standard difference
Sex, male, *n* (%)	58(55.8)	49(47.1)	17.4%	47(53.4)	38(43.2)	20.6%
Age, median (range), years	32.0(18–63)	32.0(18–67)	−1.4%	33.0(18–63)	31.0(18–67)	−11.3%
Age group, *n* (%)			2.2%			7.9%
< 40 years	76(73.1)	77(74.0)		65(73.9)	68(77.3)	
≥ 40 years	28(26.9)	27(26.0)		23(26.1)	20(22.7)	
BMI, mean ± SD, kg/m^2^	24.24 ± 4.32	24.12 ± 4.26	−2.9%	23.70 ± 3.83	23.76 ± 4.04	1.7%
Time to diagnosis of GCTB (months)	9.83 ± 26.88	9.36 ± 24.67	−1.8%	11.35 ± 26.50	9.77 ± 22.07	−6.5%
Primary tumor location, *n* (%)						
Pelvis	5(4.8)	6(5.8)	−4.3%	5(5.7)	7(8.0)	−9.0%
Spine or sacrum	11(10.6)	15(14.4)	−11.6%	32(36.4)	28(31.8)	9.6%
Upper extremity	31(29.8)	31(29.8)	0.0%	21(23.9)	23(26.1)	−5.3%
Lower extremity	57(54.8)	52(50.0)	9.6%	30(34.1)	30(34.1)	0.0%
ECOG performance status, *n* (%)			−2.0%			7.3%
0	39(37.5)	38(36.5)		27(30.7)	30(34.1)	—
≥ 1	65(62.5)	66(63.5)		61(69.3)	58(65.9)	
Metastasis, *n* (%)			0.0%			−8.8%
Yes	1(1.0)	1(1.0)		1(1.1)	2(2.3)	
No	103(99.0)	103(99.0)		87(98.9)	86(97.7)	
Relapse, *n* (%)			11.3%			−10.2%
Yes	27(26.0)	22(21.2)		22(25.0)	26(29.5)	
No	77(74.0)	82(78.8)		66(75.0)	62(70.5)	
Disease status, *n* (%)			24.4%			0.0%
Surgically challenging	104(100.0)	101(97.1)		85(96.6)	85(96.6)	
Unresectable*	0(0.0)	3(2.9)		3(3.4)	3(3.4)	
Time of administration, cycles	/	/		6.3 ± 4.18	7.3 ± 3.97	
Total dosage, mg	/	/		752.31 ± 501.60	960.29 ± 561.29	

*Note:* Data are presented as *n* (%) unless otherwise specified.

Abbreviations: BMI, body mass index; ECOG, eastern cooperative oncology group; GCTB, Giant cell tumor of bone.

* Resection could not be done without nerve damage or substantial impairment of joint function.

### 3.2 Propensity Score Analysis

The consideration of key covariates in our study was based on the clinical relevance of the management of GCTB. Of 15 covariates evaluated based on the guideline and clinical practice experience of the experts, 9 covariates (Figure S1) were included for consideration for the clinical relevance of the management of GCTB patients. After the use of directed acyclic graphs, 6 covariates were finalized in our study, which included age (years), primary tumor location, metastasis (yes or no), relapse (yes or no), ECOG performance score (0, ≥ 1) and disease duration of GCTB (months).

**FIGURE 2:**
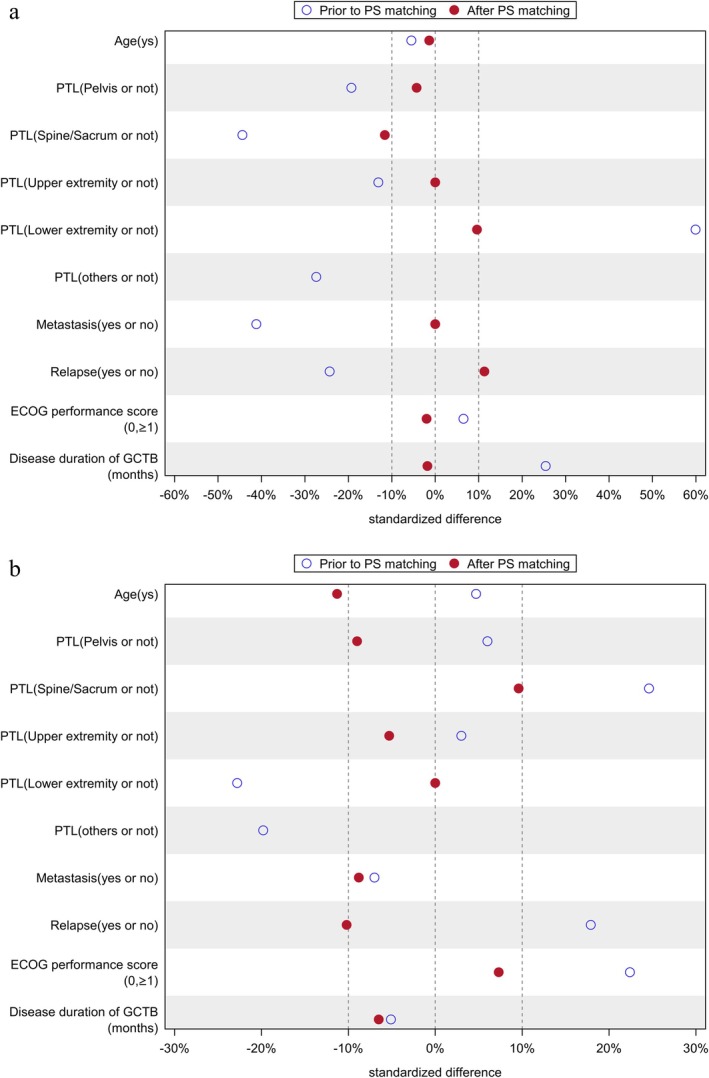
Standard difference distribution of covariates prior to and after propensity score matching. a. JMT103 vs. non‐denosumab treatment cohort. Standardized differences in most baseline covariates were less than 10% except primary tumor location (11.6%) and relapse (11.3%), indicating a good balance between cohorts. b. JMT103 vs. denosumab treatment cohort. Standardized differences in most baseline covariates were less than 10% except age (11.3%) and relapse (10.2%), indicating a good balance between cohorts. PS, Propensity score; PTL, Primary tumor location; GCTB, Giant cell tumor of bone.

After 1:1 propensity score matching, 104 patients per JMT103 and non‐denosumab cohort were included in the primary analysis. The demographic factors were similar in both cohorts, and the median age was 32 years for both cohorts; 47.1% of the JMT103 patients were male with a mean BMI of 24.12 kg/m^2^, 55.8% of the non‐denosumab patients were male with a mean BMI of 24.24 kg/m^2^ (Table 1). Standardized differences in most baseline covariates were less than 10% except for primary tumor location (11.6%) and relapse (11.3%), indicating a good balance between cohorts (Figure 2a).

After 1:1 propensity score matching, 88 patients per JMT103 and denosumab cohort were included in the primary analysis. The demographic factors were similar in both cohorts; median age was 31 years (range 18–67) and 33 years (range 18–63) for the JMT103 and denosumab cohort, 43.2% of the JMT103 patients were male with a mean BMI of 23.76 kg/m^2^, 53.4% of the denosumab patients were male with a mean BMI of 23.70 kg/m^2^ (Table 1). Standardized differences in most baseline covariates were less than 10% except age (11.3%) and relapse (10.2%), indicating a good balance between cohorts (Figure 2b).

### 3.3 Primary Endpoint: OTR

OTR was 94.2% (98/104, 95% CI 88.0%–97.3%) for the JMT103 cohort, compared with 4.8% (5/104, 95% CI 2.1%–10.8%) for the non‐denosumab cohort; group difference was 89.4% (95% CI 80.8%–93.6%) (*p* < 0.0001), OR = 435.84 (95% CI 110.28–1722.57) (Table 2).

**TABLE 2:** Summary of primary endpoint and secondary endpoints after propensity score matching (JMT103 vs. Non‐denosumab and JMT103 vs. Denosumab).

	JMT103 vs. Non‐denosumab		JMT103 vs. Denosumab	
	JMT103 cohort (*N* = 104)	Non‐denosumab cohort (*N* = 104)	JMT103 cohort (*N* = 88)	Denosumab cohort (*N* = 88)
OTR (95% CI)^a^	94.2% (88.0, 97.3)	4.8% (2.1, 10.8)	92.0% (84.5, 96.1)	67.0% (56.7, 76.0)
Group difference (95% CI)^b^	89.4% (80.8, 93.6)		25.0% (13.3, 36.1)	
*p* ^c^	< 0.001			
OR (95% CI)^d^	323.4 (95.55, 1094.58)		5.7 (2.33, 13.86)	
Tumor response rate throughout the study (95% CI)^a^	94.2% (88.0, 97.3)	4.8% (2.1, 10.8)	94.3% (87.4, 97.5)	86.4% (77.7, 92.0)
Group difference (95% CI)^b^	89.4% (80.8, 93.6)		8.0% (−1.0, 17.2)	
*p* ^c^	< 0.001			
OR (95% CI)^d^	323.4 (95.55, 1094.58)		2.6 (0.88, 7.79)	
Objective response rate (95% CI)^a^	83.5%^f^ (75.1, 89.4)	11.1%^f^ (3.9, 28.1)	87.4%^f^ (78.8, 92.8)	80.0%^f^ (70.3, 87.1)
Group difference (95% CI)^b^	72.4% (53.5, 81.8)		7.4% (−3.8, −18.5)	
*p* ^c^	< 0.001		0.191	
OR (95% CI)^d^	40.5 (10.94, 149.69)		1.7 (0.76, 3.95)	
Disease control rate (95% CI)^a^	100.0%^f^ (96.4, 100.0)	70.4%^f^ (51.5, 84.1)	100.0%^f^ (95.8, 100.0)	98.8%^f^ (93.6, 99.8)
Group difference (95% CI)^b^	29.6% (15.4, 48.5)		1.2% (−3.2, 6.4)	
*p* ^e^	< 0.001		0.494	
OR (95% CI)^d^	> 9999.999 (< 0.001, > 9999.999)		> 9999.999 (< 0.001, > 9999.999)	

Abbreviations: CI, confidence interval; OR, odd ratio.

^a^ Wilson's method was used to construct 95%.

^b^ Newcombe's method was used to construct 95%.

^c^ Pearson χ^2^ test was used for group comparison.

^d^ Nonconditional logistic regression was used to calculate OR and 95% CI, with group as a covariate included in the model.

^e^ Fisher's exact test was used for group comparison.

^f^ The proportion was calculated using the patients who had baseline and at least 1 postbaseline imaginary examination per ICDS criteria as the denominator.

OTR was 92.0% (81/88, 95% CI 84.5%–96.1%) for the JMT103 cohort compared with 67.0% (59/88, 95% CI 56.7%–76.0%) for the denosumab cohort; the group difference was 25.0% (95% CI 13.3%–36.1%), OR = 6.01 (95% CI 2.43–14.86) (Table 2).

### 3.4 Sensitivity Analysis and Subgroup Analysis

Sensitivity analysis 1–2 for the primary endpoint showed that OTR of the JMT103 cohort was significantly higher than that of the non‐denosumab cohort and was comparable with that of the denosumab cohort, which was consistent or showed similar trends with the result of the propensity score matching analysis (Tables S3–S6). In sensitivity analysis 3, multiple logistic regression analysis showed that a statistical difference in OTR was observed between the non‐denosumab cohort and the JMT103 cohort (*p* < 0.001), as well as between the denosumab cohort and the JMT103 cohort (*p* < 0.001), OR was 736.23 (95% CI 196.71–2755.49) and 6.55 (95% CI 2.86–15.00), respectively (Tables S7 and S8). In sensitivity analysis 4, E‐values were 41.24 and 4.34 for JMT103 vs. non‐denosumab and JMT103 vs. denosumab, which suggested that there were fewer possibilities of observed effectiveness caused by unmeasurable or unknown factors; hence, it confirmed that the result of the propensity score matching analysis was reliable (Figure S2).

**FIGURE 3:**
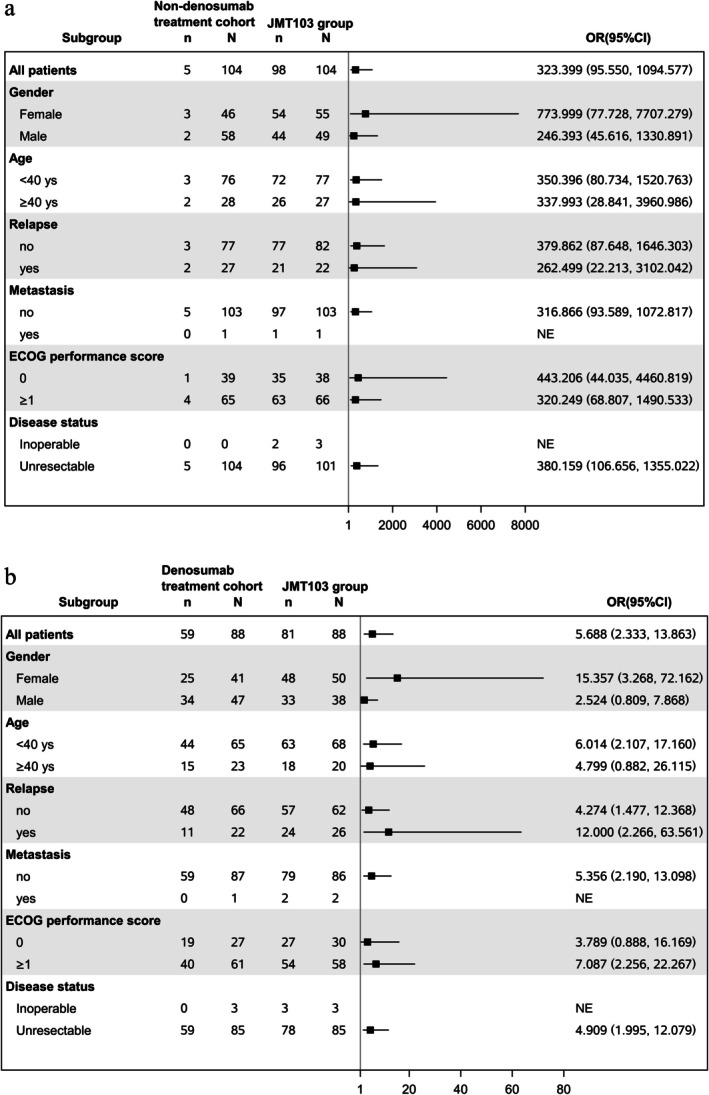
Forest plot of subgroup analysis for OTR within 12 weeks. (a) JMT103 vs. non‐denosumab cohort; (b) JMT103 vs. denosumab cohort. All the OTR within 12 weeks of the JMT103 cohort was superior to the non‐denosumab/denosumab cohort in all subgroups (Figure 3a,b).*Resection could not be done without nerve damage or substantial impairment of joint function. *N*, total number of cases; *n*, number of response cases. OTR, objective tumor response. OR, odd ratio.

In addition, OR for objective tumor response rates in all subgroups was similar to those from the propensity score matching analysis (Figure 3a,b).

### 3.5 Secondary Endpoints

The tumor response rate throughout the study was 94.2% (98/104, 95% CI 88.0%–97.3%) for the JMT103, compared with 4.8% (5/104, 95% CI 2.1%–10.8%) for the non‐denosumab cohort; the group difference was 89.4% (95% CI 80.8%–93.6%) (*p* < 0.001), OR = 323.40 (95% CI 95.55–1049.58). The tumor response rate throughout the study was 94.3% (83/88, 95% CI 87.4%–97.5%) for the JMT103, compared with 86.4% (76/88, 95% CI 77.7%–92.0%) for the denosumab cohort; the group difference was 8.0% (95% CI −1.0%–17.2%), OR = 2.62 (95% CI 0.88–7.79).

Similar results were shown in objective response rate (83.5% vs. 11.1%, 87.4% vs. 80.0%), and disease control rate (100% vs. 70.4%, 100.0% vs. 98.8%) for the JMT103 vs. non‐denosumab cohort and JMT103 vs. denosumab cohort (Table 2).

### 3.6 Safety

Of all 166 patients in the non‐denosumab treatment cohort, 29 adverse events (AEs) were recorded, one study drug‐related AE occurred, and no severe AE was observed. Of all 135 patients in the denosumab treatment cohort, 34 AEs were recorded, and 24 patients (17.8%) experienced study drug‐related AEs. Although 3 patients (2.2%) reported SAEs, none were considered to be related to denosumab treatment (Table 3). No adverse event of special interest (AESI) leading to death was observed in the non‐denosumab and denosumab treatment cohorts.

**TABLE 3:** Summary of adverse events.

	Non‐denosumab cohort (*N* = 166)		Denosumab cohort (*N* = 135)		JMT103 cohort (*N* = 139)	
	All grade	≥ Grade 3	All grade	≥ Grade 3	All grade	≥ Grade 3
AEs, *n* (%)	29 (17.5)	2 (1.2)	34 (25.2)	5 (3.7)	126 (90.6)	19 (13.7)
Treatment related AEs, *n* (%)	1 (0.6)	0	24 (17.8)	0	85 (61.2)	3 (2.2)
Common AEs (occurring > 5% of patients), *n* (%)						
Anemia	2 (1.2)	0	1 (0.7)	0	36 (25.9)	8 (5.8)
Hypophosphatemia	1 (0.6)	0	15 (11.1)	0	35 (25.2)	0
Hypocalcemia	2 (1.2)	0	9 (6.7)	0	31 (22.3)	0
ALT elevation	7 (4.2)	0	7 (5.2)	0	18 (12.9)	0
White blood cell count elevation	2 (1.2)	0	0	0	15 (10.8)	0
Upper respiratory tract infection	1 (0.6)	0	0	0	13 (9.4)	1 (0.7)
Hypermagnesemia	0	0	0	0	12 (8.6)	1 (0.7)
C‐reactive protein increased	0	0	0	0	12 (8.6)	0
Periodontal disease	0	0	0	0	12 (8.6)	0
Hypoalbuminemia	0	0	0	0	11 (7.9)	1 (0.7)
Dental caries	0	0	0	0	11 (7.9)	0
AST elevation	4 (2.4)	0	7 (5.2)	0	10 (7.2)	1 (0.7)
Weight increased	1 (0.6)	0	0	0	9 (6.5)	0
Neutrophil count elevation	0	0	0	0	8 (5.8)	0
Blood bilirubin elevation	0	0	0	0	8 (5.8)	1 (0.7)
Fever	0	0	0	0	7 (5.0)	0
Hyperuricemia	1 (0.6)	0	0	0	7 (5.0)	0
Hypomagnesaemia	0	0	0	0	7 (5.0)	2 (1.4)

Abbreviations: AE, adverse events; ALT, alanine aminotransferase; AST, aspartate aminotransferase.

In the JMT103 cohort (*n* = 139), 85 patients (61.2%) experienced study drug‐related AEs (Table 3). Although 5 patients (3.6%) were reported SAEs, none of them were considered to be related to JMT103 treatment. One AESI (grade 2 allergic dermatitis) occurred in the JMT103 cohort, which was considered to be associated with JMT103 treatment and resolved after medical intervention.

## 4 Discussion

Our study is a large‐scale study investigating the effectiveness of denosumab and non‐denosumab treatment in Chinese patients with GCTB in real‐world clinical settings. In our study, the tumor response rate was 67%–86.4% in the denosumab cohort, consistent with historical denosumab study [8, 11, 26, 27, 28]. Although the results of the JMT103 single‐arm, phase II study showed very promising results, adult patients with unresectable GCTB receiving JMT103 achieved an objective tumor response rate of 93.5% with a median time to response of 0.95 months [16], these findings suggested an improved outcome in comparison with denosumab data [8, 11, 26, 27, 28], but were limited by its single‐arm design.

In this study, we used propensity score analysis to balance the baseline characteristics between the JMT103 cohort in the single‐arm, phase II study and those receiving non‐denosumab or denosumab treatment in a real‐world setting. We also assessed the potential impact of unmeasured confounding factors on the findings (sensitivity analysis 4), which were considered to be reliable. There were some successful application [29, 30, 31] to gain US FDA approval using historical comparison to a single‐arm group, with no more than 10 covariates included in the propensity score model. In addition, denosumab real‐world study [32] in postmenopausal women with osteoporosis that included 59 covariates was a large‐scale post‐market study; it is not comparable to our study in the indication, study purpose, and study design elements.

Great progress have been made on the pathogenesis [33], diagnosis [34] and treatment [35] of GCTB, which give much opportunity for the new treatment development of GCTB. Our analyses demonstrated that JMT103 had an improvement in OTR in patients with unresectable or surgically challenging GCTB. The results were consistent across all the analyses sets and sensitivity analyses, suggesting that the results were robust. These observed outcomes may be attributed to the strategic use of an IgG4 isotype in JMT103 for the following reasons: Human IgG2 monoclonal antibodies were known to be susceptible to disulfide scrambling, which can lead to product heterogeneity during therapeutic development [36, 37]. This structural instability poses challenges for maintaining consistency in antibody‐based therapeutics [38]. As JMT103 functions as a RANKL‐blocking antibody, the use of an IgG4 isotype may offer superior safety relative to IgG2 by minimizing the formation of large immune complexes and reducing unintended effector functions [18]. Finally, JMT103 has been approved by NMPA based on the results of two pivotal clinical studies (JMT103 phase II pivotal clinical study [16] and JMT103 real‐world study [the present study]) for the treatment of unresectable or surgically challenging GCTB.

Although many challenges have been recognized to be involved in real‐world data as a comparison, considerable thought has been put into minimizing the potential biases related to selection and confounding in our study. First, the eligibility criteria of the non‐denosumab or denosumab treatment cohort were aligned with that of the single‐arm, phase II JMT103 study. Second, the real‐world non‐denosumab and denosumab data were collected from the top three hospitals where the JMT103 single‐arm study was conducted to minimize the differences in clinical management. Third, the effectiveness in real‐world non‐denosumab and denosumab treatment cohorts was assessed using the same ICDS or EORTC criteria as the single‐arm JMT103 cohort.

Our study had several limitations. First, the non‐denosumab or denosumab treatment cohort data were collected retrospectively in a real‐world setting. Data integrity and quality issues continued to be a great concern in the collection of retrospective data [39]. In our study, the data collection process has been standardized by establishing a guideline to specify the details of gathering the information, thus keeping data real, transparent, and traceable to a maximum extent. It turned out to be acceptable in the obtained data, especially the primary and key second endpoints. The missing data rates for all covariates were less than 1%, except for the ECOG score with a missing data rate of 5.0% (15/301). Second, the assessment of tumor response differed between cohorts: the JMT103 cohort used best overall response, while the real‐world denosumab cohort relied on radiological or histopathological data within 12 weeks without best overall response selection, likely leading to an underestimation of denosumab's efficacy. Considering this and the efficacy of denosumab from its registration studies [8, 11], we cautiously suggest that JMT103 has comparable antitumor activity. Third, several confounding factors might potentially affect the outcome. In our study, four sensitivity analyses were used to evaluate the impact of the unmeasured confounders in the main analysis of the objective tumor response rate for the treatment of GCTB, and the results of these prespecified analyses were consistent with those of the main analysis, which suggested that it is unlikely unmeasured confounders caused our findings and means that the results of the main analysis are robust.

In addition, due to the limitation of the retrospective real‐world study in the collection of the safety events, few AEs in non‐denosumab or denosumab treatment cohorts were recorded. And there were no study‐drug‐related severe AEs reported in the JMT103 single‐arm, phase II study [16]. This study focused on the AESI, and one AESI (grade 2 allergic dermatitis) occurred in the JMT103 cohort, while no AESI was observed in the non‐denosumab and denosumab treatment cohorts. Therefore, we cannot draw any definitive conclusion regarding the difference in safety profiles between JMT103 and denosumab treatment.

In conclusion, this study suggested that JMT103 has a better effectiveness than non‐denosumab treatment and a trend toward greater effectiveness than denosumab in adult patients with unresectable or surgically challenging GCTB. Although the present findings demonstrated the results that favor JMT103 for the treatment of GCTB, further investigation is warranted to validate the present findings. A head‐to‐head trial (NCT05813665) comparing the efficacy and safety of JMT103 and denosumab is ongoing.

## Author Contributions

N.X. and J.W.: conceptualization, methodology; H.X., Y.Z., F.W., Y.D., H.S., Y.Y., W.L., T.J., Y.L., F.T., M.L., X.H., W.Z., S.Y., L.Z., and C.T.: data curation; formal analysis; investigation; H.L.: project administration; resources; supervision; H.X.: writing – original draft; all authors: writing – review and editing.

## Disclosure

The results were presented in part as an oral presentation at the annual meeting of the Chinese Society of Clinical Oncology (CSCO) 2022.

## Ethics Statement

The study protocol was approved by the ethics committee at each study site.

## Consent

The authors have nothing to report.

## Conflicts of Interest

J.W. and H.L. are employees of CSPC Pharmaceutical Group Limited. The other authors have stated that they have no conflicts of interest.

## Supporting information


**Data S1:** Supporting Information

## Data Availability

The data that support the findings of this study are available on request from the corresponding author. The data are not publicly available due to privacy or ethical restrictions.
