# Supplementary material for: JMT103 versus Non‐Denosumab or Denosumab Treatment in Chinese Patients with Unresectable or Surgically Challenging Giant Cell Tumor of Bone: A Propensity Score‐Matched Comparison
Source: Cancer Med. 2025 Nov 25;14(22):e71340. doi: 10.1002/cam4.71340 (PMC12647920; doi:10.1002/cam4.71340)

# JMT103 versus Non-denosumab or Denosumab Treatment in Chinese Patients with Unresectable or Surgically-Challenging Giant Cell Tumor of Bone: A Propensity Score Matched Comparison

**Supplementary material: Detailed research methods**

1. Study assumption
2. Sample size determination
3. Key covariates consideration

**Supplementary Table**

Table S1. JMT103 single-arm, phase II study site list

Table S2. Demographics and clinical characteristics of patients in non-denosumab or denosumab cohort and JMT103 cohort before propensity score matching

Table S3 Sensitivity analysis 1: OTR using inverse probability weighting (JMT103 vs non-denosumab)

Table S4 Sensitivity analysis 1: OTR using inverse probability weighting (JMT103 vs denosumab)

Table S5 Sensitivity analysis 2: OTR after excluding the patients with no postbaseline histopathological evaluation and radiologic examination within 12 weeks (JMT103 vs non-denosumab)

Table S6 Sensitivity analysis 2: OTR after excluding the patients with no postbaseline histopathological evaluation and radiologic examination within 12 weeks (JMT103 vs denosumab)

Table S7 Sensitivity analysis 3: OTR using logistics regression model with adjustment of the confounders and potential heterogeneous factors (JMT103 vs non-denosumab)

Table S8 Sensitivity analysis 3: OTR using logistics regression model with adjustment of the confounders and potential heterogeneous factors (JMT103 vs denosumab)

**Supplementary Figure**

Figure S1. The screen of key covariates.

Figure S2. Sensitivity analysis 4

**Supplementary material: Detailed research methods**

1. **Study assumption**

1) Compared with non-denosumab cohort, the tumor response rate would increase remarkably in JMT103 cohort;

2) Compared with denosumab cohort, the tumor response rate would be comparable in the JMT103 cohort.

1. **Sample size determination**

The sample size was determined based on the assumption that 1) Compared with non-denosumab cohort, the tumor response rate would increase remarkably in the JMT103 cohort; 2) Compared with denosumab cohort, the tumor response rate would be comparable in the JMT103 cohort.

For model robustness, assuming the tumor response rate of 72-75% in the treatment group, eight covariates included in the model, and events per variable of 12 (normally >10 ^1^), then 96 patients without any response were needed; total sample size should be 384(96/[1-75%]) including 139 patients in JMT103 group. Thus, a sample size of 245 was required from the real-world study.

For comparing JMT103 and non-denosumab cohorts, we expected that the tumor response rate in JMT103 and non-denosumab treatment cohorts were 72% and 55%, respectively, assuming the coefficient of determination between the covariates and treatment cohorts was 0.1, then 278 of sample size would give 80% power to detect the difference in tumor response OR of 2.1 at two-side α level of 0.05. Excluding 139 patients in JMT103 cohort, 174 in the non-denosumab cohort were required, considering a 20% matching loss rate.

For comparing the JMT103 and denosumab cohort, we expected that the tumor response rate in both cohorts would be 72%, and a sample size of 105 per group would ensure the lower limit of 95% confidence interval of the tumor response rate in denosumab cohort no less than 62%. 132 in the denosumab cohort was required, considering a 20% matching loss rate.

1. **Key covariates consideration**

Of 15 covariates evaluated based on the guideline^4^ and clinical practice experience of the experts, 9 covariates (highlighted in Blue) were included for consideration for clinical relevance of the management of GCTB patients. After use of directed acyclic graphs, 6 covariates were finalized in our study, which included age (years), primary tumor location, metastasis (yes or no), relapse (yes or no), ECOG performance score (0, ≥1) and disease duration of GCTB (months) (Figure S1).

**Table S1. JMT103 single-arm, phase II study site list**

| **Site ID** | **Site Name** |
| --- | --- |
| 01 | Beijing Ji Shui Tan Hospital |
| 06 | West China Hospital of Sichuan University |
| 02 | Peking University Third Hospital |
| 09 | The First Affiliated Hospital of Sun Yat-sen University |
| 08 | Fudan University Shanghai Cancer Center |
| 24 | Sun Yat-sen University Cancer Center |
| 14 | Qilu Hospital of Shandong University |
| 16 | Liaoning Cancer Hospital & Institute |
| 21 | Hunan Cancer Hospital |
| 03 | The Fourth Medical Center of Chinese PLA General Hospital |
| 04 | The Second Affiliated Hospital of Air Force Medical University |
| 28 | Yunnan Cancer Hospital |
| 05 | Cancer Hospital Affiliated to Guangxi Medica University |
| 12 | The First Affiliated Hospital of Fujian Medical  University |
| 15 | Henan Cancer Hospital |
| 34 | Xi’an HongHui Hospital |
| 19 | The Second Affiliated Hospital Zhejiang University School of Medicine |
| 20 | The Third Hospital of Hebei Medical University |
| 17 | The Affiliated Cancer Hospital of Guizhou  Medical University |
| 23 | Tianjin Medical University Cancer Institute &  Hospital |
| 29 | Shengjing Hospital of China Medical University |
| 30 | Peking University Cancer Hospital |
| 31 | Harbin Medical University Cancer Hospital |
| 35 | Second hospital of Shanxi Medical University |

**Table S2. Demographics and clinical characteristics of patients in non-denosumab or denosumab cohort and JMT103 cohort before propensity score matching**

| **Characteristic** | **Non-denosumab cohort**  **(N = 166)** | **JMT103 cohort**  **(N = 138)** | **Standard difference** | **Denosumab cohort**  **(N = 135)** | **JMT103 cohort**  **(N = 138)** | **Standard difference** |
| --- | --- | --- | --- | --- | --- | --- |
| **Sex, male, *n*(%)** | 88(53.0) | 65(47.1) | 11.8% | 69(51.1) | 65(47.1) | 8.0% |
| **Age, median(range), years** | 31.0(18-79) | 33.0(18-67) | -5.5% | 32.0(18-63) | 33.0(18-67) | 4.7% |
| **Age group, *n*(%)** |  |  | 9.7% |  |  | 6.3% |
| **<40 years** | 118(71.1) | 104(75.4) |  | 98(72.6) | 104(75.4) |  |
| **≥40 years** | 48(28.9) | 34(24.6) |  | 37(27.4) | 34(24.6) |  |
| **BMI, mean ± SD, kg/m^2^** | 23.96±4.52 | 24.11±4.22 | 3.5% | 23.70±3.61 | 24.11±4.22 | 10.6% |
| **Time to diagnosis of GCTB (months)** | 6.77±21.97 | 13.56±30.81 | 25.4% | 15.05±27.77 | 13.56±30.81 | -5.1% |
| **Primary tumor location *n*(%)** |  |  |  |  |  |  |
| **Pelvis** | 5(3.0) | 10(7.2) | -19.2% | 12(8.9) | 10(7.2) | 0.7% |
| **Spine or sacrum** | 11(6.6) | 30(21.7) | -44.2% | 44(32.6) | 30(21.7) | 29.1% |
| **Upper extremity** | 33(19.9) | 35(25.4) | -14.4% | 36(26.7) | 35(25.4) | 4.3% |
| **Lower extremity** | 117(70.5) | 58(42.0) | 60.9% | 42(31.1) | 58(42.0) | -25.8% |
| **ECOG performance status *n*(%)** |  |  | 6.5% |  |  | 22.4% |
| **0** | 57(34.5) | 52(37.7) |  | 33(24.4) | 52(37.7) |  |
| **≥ 1** | 108(65.5) | 86(62.3) |  | 88(65.2) | 86(62.3) |  |
| **Missing** | 1(0.0) | 0(0.0) |  | 14(10.4) | 0(0.0) |  |
| **Metastasis *n*(%)** |  |  | -41.2% |  |  | -7.0% |
| **Yes** | 1(0.6) | 13(9.4) |  | 10(7.5) | 13(9.4) |  |
| **No** | 164(99.4) | 125(90.6) |  | 124(92.5) | 125(90.6) |  |
| **Missing** | 1(0.6) | 0(0.0) |  | 1(0.0) | 0(0.0) |  |
| **Relapse *n*(%)** |  |  | -24.3% |  |  | 17.9% |
| **Yes** | 29(17.5) | 38(27.5) |  | 48(35.8) | 38(27.5) |  |
| **No** | 137(82.5) | 100(72.5) |  | 86(64.2) | 100(72.5) |  |
| **Missing** | 0(0.0) | 0(0.0) |  | 1(0.0) | 0(0.0) |  |
| **Disease status *n*(%)** |  |  | 43.6% |  |  | -5.7% |
| **Inoperable** | 166(100) | 126(91.3) |  | 121(89.6) | 126(91.3) |  |
| **Unresectable*** | 0(0.0) | 12(8.7) |  | 14(10.4) | 12(8.7) |  |
| **Previous therapy *n*(%)** |  |  |  |  |  |  |
| **Surgery** | 166 (100) | 43 (31.2) |  | 120 (88.9) | 43 (31.2) |  |
| **Bisphosphonates** | 21 (12.7) | 6 (4.3) |  | 0 (0.0) | 6 (4.3) |  |
| **Radiation therapy** | 0 (0.0) | 0 (0.0) |  | 0 (0.0) | 0 (0.0) |  |

Data are presented as n (%) unless otherwise specified.

*Resection could not be done without nerve damage or substantial impairment of joint function.

BMI, body mass index; ECOG, eastern cooperative oncology group; GCTB, Giant cell tumor of bone.

**Table S3 Sensitivity analysis 1: OTR using inverse probability weighting (JMT103 vs non-denosumab)**

|  | |  | | Non-denosumab cohort(N = 166) | JMT103 cohort(N = 138) |
| --- | --- | --- | --- | --- | --- |
| Investigator/IRC | | Tumor response |  |  | |
|  |  |  | 5.4/138.6 | 128.0/137.0 | |
|  |  | Tumor response rate (95%CI) ^a^ | 3.9% (1.7%, 8.5%) | 93.4% (88.0%, 96.5%) | |
|  |  | Group difference (95%CI) ^b^ |  | 89.5% (82.4%, 93.3%) | |
|  |  | Statistics/*P* value ^c^ |  | 221.173/<0.001 | |
|  |  | OR (95%CI) ^d^ |  | 351.435 (117.606, 1050.176) | |

Abbreviations: IRC, independent review committee; CI, confidence interval; OR, odd ratio.

^a^ Wilson’s method was used to construct 95%CI;

^b^ Newcombe’s method was used to construct 95%CI;

^c^ Pearson χ^2^ test was used for group comparison;

^d^ Non-conditional logistic regression was used to calculate OR and 95%CI, with group as covariate included in the model.

**Table S4 Sensitivity analysis 1: OTR using inverse probability weighting (JMT103 vs denosumab)**

|  | |  | | Denosumabcohort(N = 135) | JMT103 cohort(N = 138) |
| --- | --- | --- | --- | --- | --- |
| Investigator/IRC | | Tumor response |  |  | |
|  |  |  | 113.3/147.4 | 128.0/137.0 | |
|  |  | Tumor response rate (95%CI) ^a^ | 76.9% (69.4%, 83.0%) | 93.4% (88.0%, 96.5%) | |
|  |  | Group difference (95%CI) ^b^ |  | 16.5% (8.4%, 24.6%) | |
|  |  | OR (95%CI) ^c^ |  | 4.275 (1.966, 9.296) | |

Abbreviations: IRC, independent review committee; CI, confidence interval; OR, odd ratio.

^a^ Wilson’s method was used to construct 95%CI;

^b^ Newcombe’s method was used to construct 95%CI;

^c^ Non-conditional logistic regression was used to calculate OR and 95%CI, with group as covariate included in the model.

**Table S5 Sensitivity analysis 2: OTR after excluding the patients with no postbaseline histopathological evaluation and radiologic examination within 12 weeks (JMT103 vs non-denosumab)**

|  | |  | | Non-denosumab cohort(N = 103) | JMT103 cohort(N = 103) |
| --- | --- | --- | --- | --- | --- |
| Investigator/IRC | | Tumor response |  |  | |
|  |  |  | 5/103 | 99/103 | |
|  |  | Tumor response rate (95%CI) ^a^ | 4.9% (2.1%, 10.9%) | 96.1% (90.4%, 98.5%) | |
|  |  | Group difference (95%CI) ^b^ |  | 91.3% (83.0%, 94.9%) | |
|  |  | Statistics/*P* value ^c^ |  | 171.589/<0.001 | |
|  |  | OR (95%CI) ^d^ |  | 485.093 (126.498, 1860.230) | |

Abbreviations: IRC, independent review committee; CI, confidence interval; OR, odd ratio.

^a^ Wilson’s method was used to construct 95%CI;

^b^ Newcombe’s method was used to construct 95%CI;

^c^ Pearson χ^2^ test was used for group comparison;

^d^ Non-conditional logistic regression was used to calculate OR and 95%CI, with group as covariate included in the model.

**Table S6 Sensitivity analysis 2: OTR after excluding the patients with no postbaseline histopathological evaluation and radiologic examination within 12 weeks (JMT103 vs denosumab)**

|  | |  | | Denosumabcohort(N = 84) | JMT103 cohort(N = 84) |
| --- | --- | --- | --- | --- | --- |
| Investigator/IRC | | Tumor response |  |  | |
|  |  |  | 71/84 | 77/84 | |
|  |  | Tumor response rate (95%CI) ^a^ | 84.5% (75.3%, 90.7%) | 91.7% (83.8%, 95.9%) | |
|  |  | Group difference (95%CI) ^b^ |  | 7.1% (-2.9%, 17.3%) | |
|  |  | OR (95%CI) ^c^ |  | 2.014 (0.761, 5.333) | |

Abbreviations: IRC, independent review committee; CI, confidence interval; OR, odd ratio.

^a^ Wilson’s method was used to construct 95%CI;

^b^ Newcombe’s method was used to construct 95%CI;

^c^ Non-conditional logistic regression was used to calculate OR and 95%CI, with group as covariate included in the model.

**Table S7 Sensitivity analysis 3: OTR using logistics regression model with adjustment of the confounders and potential heterogeneous factors (JMT103 vs non-denosumab)**

|  | Estimator | OR (95%CI) | Statistics | *p* value |
| --- | --- | --- | --- | --- |
| Cohort |  |  |  |  |
| JMT103 | 6.602 | 736.23(196.71, 2755.49) | 96.11 | <0.001 |
| Non-denosumab | Reference |  |  |  |
| Sex |  |  |  |  |
| Male | -0.674 | 0.510(0.156, 1.664) | 1.246 | 0.264 |
| Female | Reference |  |  |  |
| Age(years) | -0.012 | 0.988(0.938, 1.040) | 0.221 | 0.638 |
| Metastasis |  |  |  |  |
| Yes | -0.361 | 0.697(0.071, 6.811) | 0.096 | 0.756 |
| No | Reference |  |  |  |
| Relapse |  |  |  |  |
| Yes | -1.713 | 0.180(0.037, 0.870) | 4.551 | 0.033 |
| No | Reference |  |  |  |
| ECOG performance score |  |  |  |  |
| ≥1 | 0.759 | 2.136(0.639, 7.145) | 1.518 | 0.218 |
| 0 | Reference |  |  |  |
| Time to diagnosis of GCTB (month) | -0.027 | 0.973(0.957, 0.989) | 10.365 | 0.001 |

**Table S8 Sensitivity analysis 3: OTR using logistics regression model with adjustment of the confounders and potential heterogeneous factors (JMT103 vs denosumab)**

|  | Estimator | OR (95%CI) | Statistics | *p* value |
| --- | --- | --- | --- | --- |
| Cohort |  |  |  |  |
| JMT103 | 1.879 | 6.545(2.855, 15.003) | 19.706 | <0.001 |
| Denosumab | Reference |  |  |  |
| Sex |  |  |  |  |
| Male | -0.310 | 0.733(0.362, 1.485) | 0.742 | 0.389 |
| Female | Reference |  |  |  |
| Age(years) | 0.002 | 1.002(0.969, 1.037) | 0.020 | 0.888 |
| Metastasis |  |  |  |  |
| Yes | 0.229 | 1.257(0.243, 6.493) | 0.075 | 0.784 |
| No | Reference |  |  |  |
| Relapse |  |  |  |  |
| Yes | 0.242 | 1.274(0.488, 3.330) | 0.244 | 0.621 |
| No | Reference |  |  |  |
| ECOG performance score |  |  |  |  |
| ≥1 | -0.140 | 0.870(0.393, 1.926) | 0.118 | 0.731 |
| 0 | Reference |  |  |  |
| Time to diagnosis of GCTB (month) | -0.013 | 0.987(0.975, 1.000) | 3.961 | 0.047 |

**Figure S1 The screen of key covariates.**

**
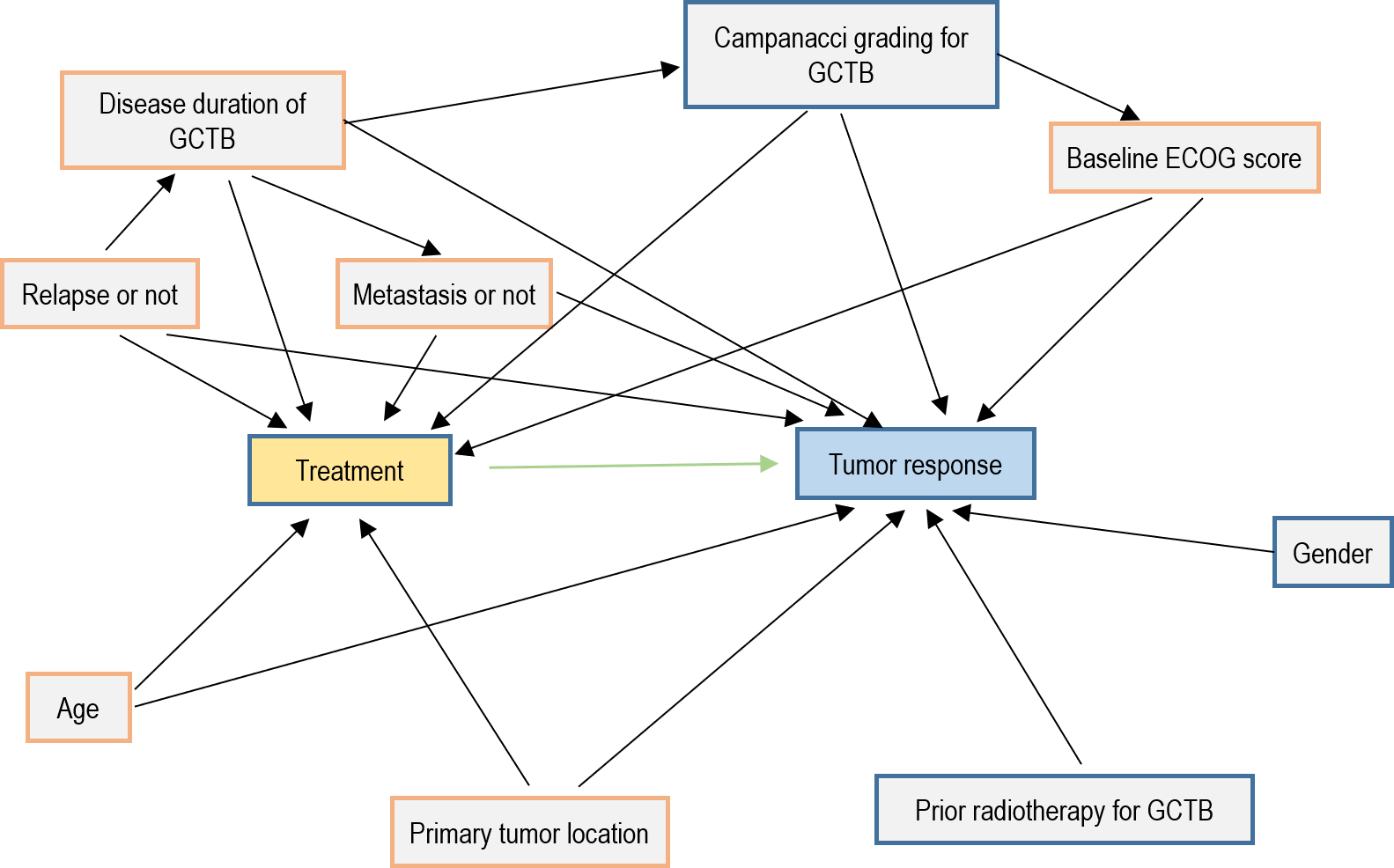
**

**Figure S2 Sensitivity analysis 4**


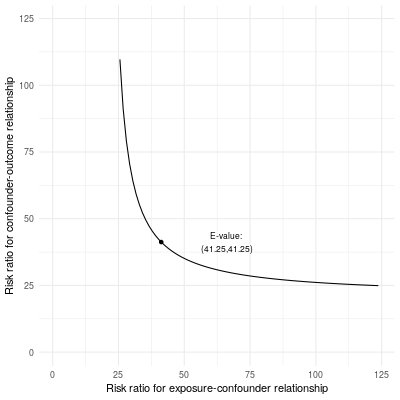

Supplement: Supplementary file 1 — Data S1: Supporting Information [file CAM4-14-e71340-s001.docx]
